# Histogram analysis comparison of readout-segmented and single-shot echo-planar imaging for differentiating luminal from non-luminal breast cancer

**DOI:** 10.1038/s41598-024-62514-0

**Published:** 2024-05-27

**Authors:** Yiqi Hu, Qilan Hu, Zhiqiang Liu, Cicheng Huang, Liming Xia

**Affiliations:** 1 Department of Radiology, Tongji Hospital, Tongji Medical College, Huazhong University of Science and Technology, Wuhan, 430030 Hubei China; 2 Center of Stomatology, Tongji Hospital, Tongji Medical College, Huazhong University of Science and Technology, Wuhan, 430030 Hubei China

**Keywords:** Magnetic resonance imaging, Ss-EPI, Rs-EPI, Luminal, Non-luminal, Cancer, Breast cancer, Cancer imaging

## Abstract

To compare diffusion-kurtosis imaging (DKI) and diffusion-weighted imaging (DWI) parameters of single-shot echo-planar imaging (ss-EPI) and readout-segmented echo-planar imaging (rs-EPI) in the differentiation of luminal vs. non-luminal breast cancer using histogram analysis. One hundred and sixty women with 111 luminal and 49 non-luminal breast lesions were enrolled in this study. All patients underwent ss-EPI and rs-EPI sequences on a 3.0T scanner. Histogram metrics were derived from mean kurtosis (MK), mean diffusion (MD) and the apparent diffusion coefficient (ADC) maps of two DWI sequences respectively. Student’s t test or Mann–Whitney U test was performed for differentiating luminal subtype from non-luminal subtype. The ROC curves were plotted for evaluating the diagnostic performances of significant histogram metrics in differentiating luminal from non-luminal BC. The histogram metrics MK_mean_, MK_50th_, MK_75th_ of luminal BC were significantly higher than those of non-luminal BC for both two DWI sequences (all P<0.05). Histogram metrics from rs-EPI sequence had better diagnostic performance in differentiating luminal from non-Luminal breast cancer compared to those from ss-EPI sequence. MK_75th_ derived from rs-EPI sequence was the most valuable single metric (AUC, 0.891; sensitivity, 78.4%; specificity, 87.8%) for differentiating luminal from non-luminal BC among all the histogram metrics. Histogram metrics of MK derived from rs-EPI yielded better diagnostic performance for distinguishing luminal from non-luminal BC than that from ss-EPI. MK_75th_ was the most valuable metric among all the histogram metrics.

## Introduction

Breast cancer (BC), a disease of high heterogeneity, is divided into four molecular subtypes (luminal A, luminal B, human epidermal growth factor receptor 2- (HER2-) enriched, and triple negative) according to immunohistochemistry (IHC) markers, such as receptor expression of estrogen (ER) and progesterone (PR), HER-2 expression status, and Ki67 index ^1^. These subtypes make up an uneven proportion of breast cancer patients and behave differently in therapeutic response, metastatic patterns and prognosis^2,3^. Generally, luminal subtype (luminal A and B) accounts for the largest proportion (around 70%) of all breast cancers^4^. Luminal subtype is hormone-receptor positive thus responds best to endocrine therapy (ET) (luminal A) or combined chemotherapy and ET (luminal B)^5–7^. Non-luminal subtype including HER2-enriched and triple negative breast cancer is more aggressive and more likely to suffer from more frequent recurrence and metastasis as well as poorer prognosis when compared with luminal subtype^8^. HER2-enriched type benefits from targeted antibody therapy such as trastuzumab (Herceptin). Triple-negative breast cancer which is negative for ER/PR/HER2, shows no response to endocrine therapy or trastuzumab (Herceptin) and conventional cytostatic chemotherapy remains the only therapeutic option^9^.

The heterogeneity of the BC subtypes (luminal vs. non luminal) would affect clinical efforts to manage treatment measures and anticipate risks^10^. Therefore, there is an urgent need for accurate preoperative differentiation between luminal and non-luminal subtypes. The BC subtype classification before surgery is mainly based on breast biopsy by now. However, there exists a discordance between the biopsies and postoperative pathological diagnoses due to sampling bias^11^. Currently, MR imaging such as contrast-enhanced imaging and diffusion-weighted imaging (DWI) has been proven to provide the entire tumor information about the molecular subtype characterization^12,13^.

DWI as a non-contrast MRI technique can provide additional information on tumor characteristics and heterogeneity by calculating quantitative parameters derived from different quantitative models, such as a mono-exponential model and diffusion-kurtosis imaging (DKI)^14^. Breast DWI images are usually acquired with single-shot echo-planar imaging (ss-EPI) or readout-segmented echo-planar imaging (rs-EPI)^15^. While the motion-insensitivity and speed of ss-EPI currently preserve its role as the standard clinical DWI technique for breast, it suffers from susceptibility artifacts, geometric distortions, signal-intensity dropout, and T2* induced blurring, primarily due to slow traversal through k-space along the phase-encoding direction^16,17^. The rs-EPI sequence provides an alternative method for improved speed, resolution, or image quality by sampling a subset of k-space points in the readout direction at each shot as a means of reducing distortion and blurring at the expense of longer scan time and some navigator-correction^18,19^. However, breast DWI recommended technique has not yet been fully established in clinical application. In recent years, certain studies have attempted to figure out the correlations between DWI or DKI parameters and tumor subtype^7,20–22^. Despite several attempts to standardize imaging markers, there is still diversity in DWI scanning techniques and DWI models, which most likely contributed to DWI assessment's low capacity to distinguish between BC molecular subtypes.

Recent studies have suggested that histogram analysis may be able to more accurately assess the intratumor heterogeneity and aggressiveness of BC^23,24^. The aim of our research was to compare the histogram metrics of rs-EPI and ss-EPI sequences for differentiating luminal from non-Luminal BC.

## Materials and methods

### Patients

This prospective, unicentric study was approved by the local Institutional Review Board of Tongji Hospital, Tongji Medical College, Huazhong University of Science and Technology, and written informed consent was obtained from all participating subjects. It complies with the ethical principles of the Declaration of Helsinki. From December 2018 to December 2019, 194 women who met the inclusion criteria were selected for breast MRI in our institution database. The following inclusion criteria were used: (1) suspicious lesions that diagnosed as Breast Imaging Reporting and Data System (BI-RADS) category 4 or 5 at mammography or ultrasonography; (2) complete breast MRI scanning; (3) underwent surgery for lesion excision within one week following the MRI.

The exclusion criteria consisted of the following: (1) severe MRI susceptibility or motion artifacts (*n* = 5); (2) non-mass like lesions (*n*=11); (3) the largest size of lesions< 1cm (*n* = 6); (4) biopsy or breast-related treatment such as radiotherapy and chemotherapy for breast lesion before MRI (*n* = 9); (5) no pathological findings reported invasive ductal carcinoma (IDC) (*n* = 3).

### MR imaging

Breast MR imaging was performed using a 3.0-tesla MR scanner (Skyra, Siemens Healthineers, Erlangen, Germany) with a breast bilateral 16-channel phased-array coil. Images were obtained using the following sequences:

- Axial T2-weighted MR imaging (T2WI) with fat saturation: repetition time (TR) = 3700ms, echo time (TE) = 101ms, field of view (FOV) = 320 x 320 mm2, matrix = 224 × 320, slice thickness = 4.0 mm, voxel size = 1.5 × 1.5 × 5 mm, acquisition time (TA) = 2min6sec;
- Axial ss-EPI with fat saturation: TR = 5000 ms, TE = 96 ms, FOV = 169 × 280 mm2, matrix = 114 × 188, slice thickness = 5.0 mm, voxel size = 1.5 × 1.5 × 5 mm, averages = 5; TA =4 min 35 sec, 4 different b factors (0, 50, 1000, and 2000 s/mm2);
- Axial rs-EPI with fat saturation: TR = 5000 ms, TE = 68 ms, FOV = 169 × 280 mm2, matrix = 114 × 188, slice thickness = 5.0 mm, voxel size = 1.5 × 1.5 × 5 mm, averages = 1, readout segments = 5, TA = 4 min 27 sec, 4 different b factors (0, 50, 1000, and 2000 s/mm2);
- Axial T1-weighted dynamic contrast-enhanced magnetic resonance imaging (DCE-MRI): TR = 5.40ms, TE = 2.46ms, flip angle = 10°, FOV = 320 x 320 mm2, matrix = 243 × 320, slice thickness = 1.5mm, temporal resolution = 11.2s/phase, TA = 5min57sec. The contrast material (Omniscan, GE Healthcare, Milwaukee, WI, USA) was administrated intravenously using an automated injector with a dose of 0.1mmol/kg of body weight at an rate of 2.5 ml/ sec, which was followed by a 20-ml saline flush.

### Image analysis

All DWI imaging data were postprocessed using the prototype software, Body Diffusion Toolbox (version 0.2.2, Siemens Healthcare)^25^. Two radiologists (Z.C.A. and H.Y.Q. with 5 and 9 years of experience in breast MR imaging, respectively) who recognized the IDC diagnosis but were blinded to DWI image sequence information and the detailed histological results, reviewed the MR images. The processing workflow of DWI images consisted of the following three steps:

- Images loading. The DWI images were loaded to the software.
- Regions of interest (ROI) drawing. The largest lesion (avoiding obvious calcifications, necrosis, and vessels) was selected by consensus with matched images on T2WI and DCE-MRI as references. ROI was freehand defined around mass on the ss-EPI images with a b value of 1000 sec/mm2 and copied to the corresponding parametric maps (MK, MD and ADC). In the meantime, the ROI was saved as a mask file, which can be loaded onto the rs-EPI image within the software.
- Histogram analysis. The mathematical quotation of calculating ADC value based on mono-exponential model was as follows: $\mathit{ln}\left[S\left(b\right)\right]=\mathit{ln}\left[S\left(0\right)\right]-bADC$ ^26^. Where S(b) and S(0) are the signal intensity at a certain b value of 1000 sec/mm2 and 0 respectively. The mathematical quotation of calculating MK and MD value based on DKI model was as follows:$\text{ln}\left[S\left(b\right)\right]=\text{ln}\left[S\left(0\right)\right]-bMD+{ b}^{2}M{D}^{2}MK+O\left({b}^{3}\right)$ ^27^. Where Sb is the signal intensities of 4 b-values (0, 50, 1000, and 2000 sec/mm2). O(b3) is the fit error. Previous studies suggested that more than three b-values including at least two b-values both above and below 1000 s/mm2, to facilitate the successful capture of the non-Gaussian behavior^28,29^. Histogram metrics were extracted from each ROI of the parametric maps, which included mean, the 25, 50th, 75th and 100th percentiles, skewness and kurtosis values.
- The readers repeated all quantitative measurements after a time interval of 2 weeks.

### Histopathologic analysis

ER and PR positivity were defined by more than 1% of the nuclei stained positive in ten high-power fields. HER2 was considered positive with an IHC score of 3+ or fluorescence in situ hybridization (FISH) amplification with a ratio ≥ 2.0. According to the expression status of ER, PR, HER2, and Ki-67, the tumor was classified as Luminal A (ER and/or PR positive, HER2 negative, and Ki-67 < 14%), Luminal B (ER and/or PR positive, HER2 negative, and Ki-67 ≥ 14% or ER and/or PR positive, HER2 positive), HER2-enriched (ER and PR negative, HER2 positive), and TN (ER negative, PR negative, and HER2 negative)^3^.

### Statistical analysis

Statistical analyses were performed using SPSS (version 20.0; SPSS, Inc, Chicago, IL, USA). The intraclass correlation coefficient (ICC) was calculated for histogram metrics derived from ss-EPI and rs-EPI to evaluate inter and intra-reader agreement. The agreement, with an ICC ranging from 0 to 0.40, was considered poor, 0.40–0.59 was defined as fair, 0.60–0.74 was deemed as good, 0.75–1.00 was indicated excellent^30^. The measurements of one random radiologist were used for subsequent statistical analyses.

The student’s t test when normally distributed or Mann-Whitney U test when not normally distributed was used for the comparison of each histogram metric between ss-EPI and rs-EPI for luminal and non-luminal BC, respectively, and the differences of each histogram metric between luminal and non-luminal BC in the respective sequence. The ROC curves were constructed to compare differences in diagnostic performance between two sequences for the differentiation of luminal subtype from the other subtype. Then the sensitivity and specificity for histogram metrics were obtained at the threshold values. The area under the receiver operating characteristic (AUC) was calculated and compared between ss-EPI and rs-EPI. For all tests, *p* value less than 0.05 was considered statistically significant.

### Informed consent

Written informed consent was obtained from all participating subjects

## Results

### Demographics

A total of 160 patients (mean age ± SD, 44.3± 17.2 years; age range, 24-67 years) with 160 lesions (mean diameter, 1.53 ± 0.71 cm) were finally enrolled in our study cohort. There were 35 (21.9%) luminal A, 76 (47.5%) luminal B, 29 (18%) HER2-enriched, and 20 (12.5%) triple negative tumors.

### Comparison of Histogram metrics between ss-EPI and rs-EPI sequence

**Figure 1:**
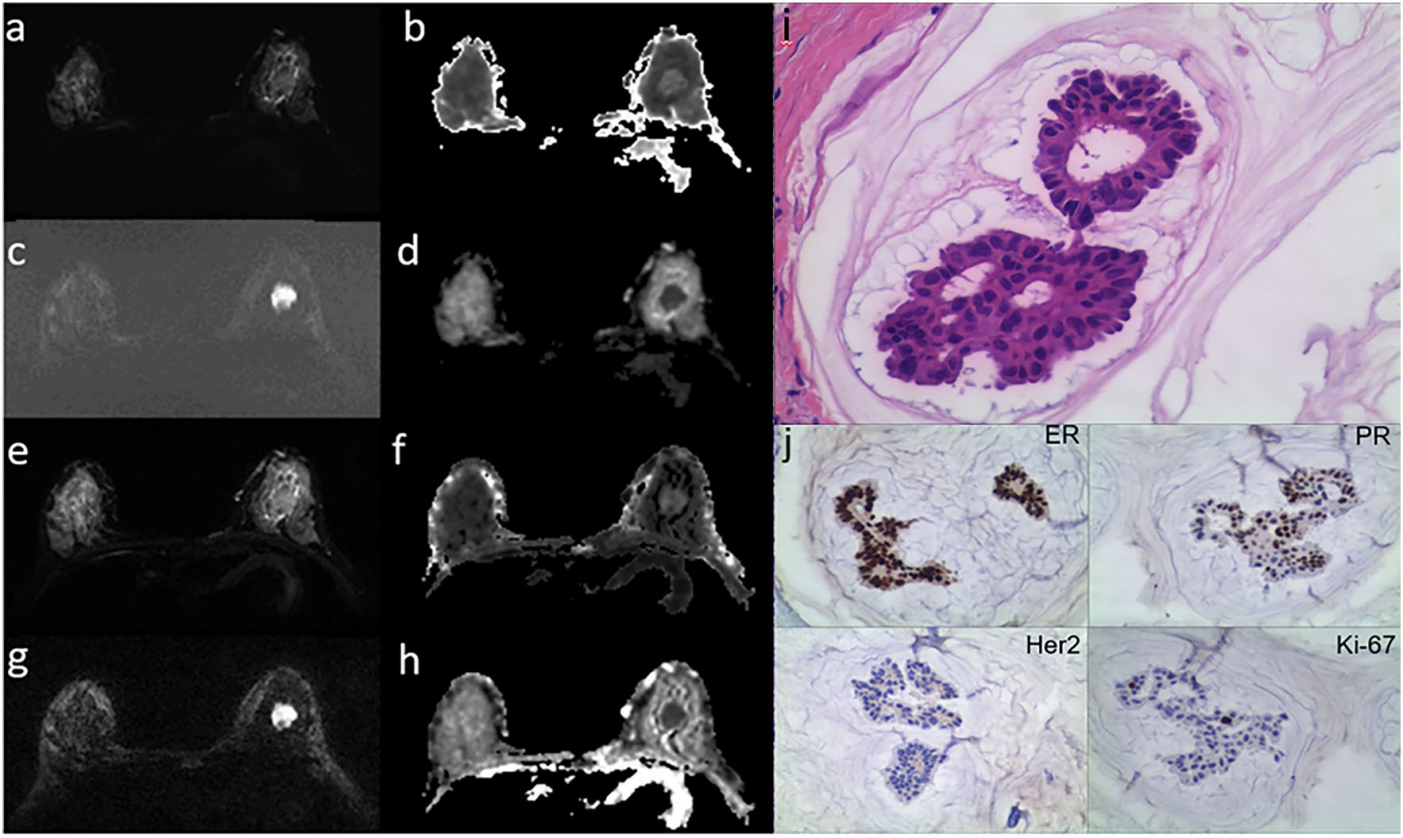
Representative images of a 33-year-old patient with luminal A type breast cancer (**a**–**j**). Ss-EPI with DWI image (**a**); MK image (**b**) with MK_mean_: 1.54, MK_25th_: 1.44, MK_50th_: 1.56, MK_75th_:1.66, MK_100th_: 2.03, MK_skewness_: −0.50, MK_kurtosis_: 3.65; MD image (**c**) with MD_mean_: 0.72×10^-3^mm2/s, MD_25th_: 0.57×10^-3^mm2/s, MD_50th_: 0.66×10^-3^mm2/s, MD_75th_: 0.79×10^-3^mm2/s,MD_100th_: 1.58×10^-3^mm2/s, MD_skewness_: 1.75×10^-3^mm2/s, MD_kurtosis_: 6.87×10^-3^mm2/s; and ADC image (**d**) with ADC_mean_: 0.49×10^-3^mm2/s, ADC_25th_: 0.42×10^-3^mm2/s, ADC_50th_: 0.45×10^-3^mm2/s, ADC_75th_: 0.52×10^-3^mm2/s, ADC_100th_: 0.92×10^-3^mm2/s, ADC_skewness_: 1.70×10^-3^mm2/s, ADC_kurtosis_: 6.71×10^-3^mm2/s. Rs-EPI with DWI image (**e**); MK image (**f**) with MK_mean_: 1.28, MK_25th_: 1.13, MK_50th_: 1.30, MK_75th_: 1.43, MK_100th_: 1.79, MK_skewness_: −0.36, MK_kurtosis_: 2.30; MD image (**g**) with MD_mean_: 0.70×10^-3^mm2/s, MD_25th_: 0.59×10^-3^mm2/s, MD_50th_: 0.69×10^-3^mm2/s, MD_75th_: 0.93×10^-3^mm2/s, MD_100th_: 1.68×10^-3^mm2/s, MD_skewness_: 0.99×10^-3^mm2/s, MD_kurtosis_: 3.91×10^-3^mm2/s; and ADC image (**h**) with ADC_mean_: 0.53×10^-3^mm2/s, ADC_25th_: 0.45×10^-3^mm2/s, ADC_50th_: 0.51×10^-3^mm2/s, ADC_75th_: 0.61×10^-3^mm2/s, ADC_100th_: 0.87×10^-3^mm2/s, ADC_skewness_: 0.74×10^-3^mm2/s, ADC_kurtosis_: 2.92×10^-3^mm2/s. Hematoxylin and eosin staining map (×200, **i**) and Immunohistochemical map (×200, **j**).

**Figure 2:**
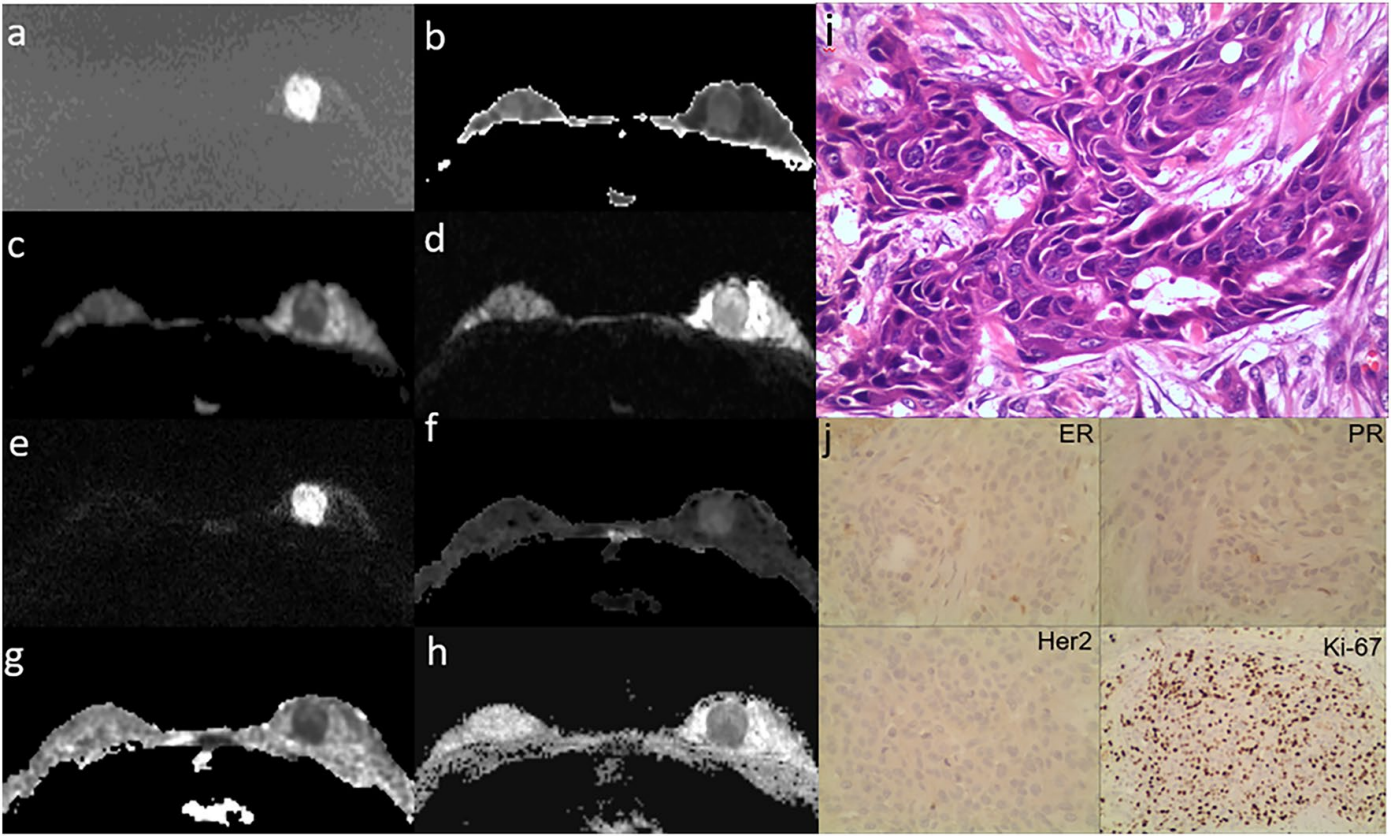
Representative images of a 41-year-old patient with HER2-enriched type breast cancer (**a**–**j**). Ss-EPI with DWI image (**a**); MK image (**b**) with MK_mean_: 0.92, MK_25th_: 0.86, MK_50th_: 0.96, MK_75th_: 1.10, MK_100th_: 1.12, MK_skewness_: −1.07, MK_kurtosis_: 3.47; MD image (**c**) with MD_mean_: 1.30×10^-3^mm2/s, MD_25th_: 1.19×10^-3^mm2/s, MD_50th_: 1.30×10^-3^mm2/s, MD_75th_: 1.41×10^-3^mm2/s, MD_100th_: 1.73×10^-3^mm2/s, MD_skewness_: −0.15×10^-3^mm2/s, MD_kurtosis_: 6.46×10^-3^mm2/s; and ADC image (**d**) with ADC_mean_: 0.70×10^-3^mm2/s, ADC_25th_: 0.63×10^-3^mm2/s, ADC_50th_: 0.66×10^-3^mm2/s, ADC_75th_: 0.75×10^-3^mm2/s, ADC_100th_: 0.92×10^-3^mm^2^/s, ADC_skewness_: 0.82×10^-3^mm^2^/s, ADC_kurtosis_: 2.64×10^-3^mm2/s. Rs-EPI with DWI image (**e**); MK image (**f**) with MK_mean_: 0.67, MK_25th_: 0.60, MK_50th_: 0.67, MK_75th_: 0.72, MK_100th_: 0.85, MK_skewness_: −0.01, MK_kurtosis_: 2.59; MD image (**g**) with MD_mean_: 1.38, MD_25th_: 1.24, MD_50th_: 1.33, MD_75th_: 1.47, MD_100th_: 2.15, MD_skewness_: 1.43, MD_kurtosis_: 5.15; and ADC image (**h**) with ADC_mean_: 0.77×10^-3^mm2/s, ADC_25th_: 0.72×10^-3^mm2/s, ADC_50th_: 0.74×10^-3^mm2/s, ADC_75th_: 0.79×10^-3^mm2/s, ADC_100th_: 1.24×10^-3^mm2/s, ADC_skewness_: 2.31×10^-3^mm2/s, ADC_kurtosis_: 9.85×10^-3^mm2/s. Hematoxylin and eosin staining map (×200, **i**) and Immunohistochemical map (×200, **j**).

Good or excellent agreements for inter- and intra-observers were exhibited in most histogram metrics derived from ss-EPI and rs-EPI sequence (Table 1). The mean and 95% CI of each histogram metric for luminal and non-luminal lesions are listed in Tables 1 and 2. The histogram metrics MK_mean_, MK_50th_, MK_75th_, MK_100th_ of luminal BC were significantly higher than those of non-luminal BC in the rs-EPI sequence (*P* = 0.003, 0.008, 0.002 and 0.001, respectively). The histogram metrics MK_mean_, MK_50th_ and MK_75th_ of luminal BC were significantly higher than those of non-luminal BC (*P* = 0.032,0.032 and 0.044, respectively) in the ss-EPI sequence. We did not find any significant differences in the histogram metrics derived from MD and ADC between luminal and non-luminal BC in both two sequences (all *P* > 0.05).The luminal BC displayed statistical differences in the MK_mean_, MK_50th_ and MK_75th_ between ss-EPI and rs-EPI sequence (*P* = 0.014, 0.045 and 0.044, respectively). The non-luminal BC showed significant differences in the MK_mean_, MK_25th_, MK_50th_ and MK_75th_ between ss-EPI and rs-EPI sequence (*P* = 0.000, 0.047, 0.023 and 0.020, respectively). The luminal and non-luminal BC showed no significant differences between two sequences for each histogram metric of MD and ADC (all *P* > 0.05). Representative images of luminal and non-luminal BC were shown in Figures 1 and 2.

**Table 1:** Comparisons of the histogram parameters between luminal and non-luminal BC in ss-EPI and rs-EPI sequence.

Parameters	Histogram Metrics	Luminal BC	Non-luminal BC	Intraobserver ICC	Interobserver ICC	*P*
Rs-EPI MK	Mean	0.96 ± 0.16	0.80 ± 0.08	0.988 (0.982,0.990)	0.974 (0.965,0.981)	0.003
	25th	0.85 ± 0.16	0.72 ± 0.10	0.925 (0.899,0.945)	0.931 (0.907,0.949)	0.083
	50th	0.96 ± 0.16	0.81 ± 0.08	0.978 (0.970,0.984)	0.972 (0.962,0.979)	0.008
	75th↵	1.08 ± 0.20	0.88 ± 0.07	0.983 (0.977,0.988)	0.985 (0.980,0.989)	0.002
	100th	1.46 ± 0.56	1.11 ± 0.22	0.918 (0.890,0.939)	0.991 (0.988,0.993)	0.001
	Skewness	0.02 ± 1.10	0.28 ± 1.13	0.662 (0.565,0.741)	0.894 (0.857,0.921)	0.594
	Kurtosis	4.60 ± 4.14	4.92 ± 4.13	0.767 (0.695,0.824)	0.615 (0.508,0.703)	0.687
MD(×10^-3^mm2/s)	Mean	1.12 ± 0.20	1.26 ± 0.29	0.970 (0.959,0.978)	0.975 (0.966,0.982)	0.990
	25th	0.98 ± 0.19	1.11 ± 0.17	0.975 (0.966,0.982)	0.973 (0.963,0.980)	0.897
	50the	1.09+0.20	1.22 ± 0.27	0.984 (0.894,0.942)	0.981 (0.979,0.989)	0.557
	75th	1.23 ± 0.23	1.38 ± 0.21	0.928 (0.903,0.947)	0.961 (0.948,0.972)	0.628
	100th	1.78 ± 0.38	1.98 ± 045	0.732 (0.651,0.797)	0.765 (0.692,0.822)	0.172
	Skewness	0.80 ± 0.69	0.85 ± 0.72	0.675 (0.581,0.751)	0.773 (0.702,0.829)	0.853
	Kurtosis	4.11 ± 1.89	4.25 ± 1.67	0.597 (0.487,0.688)	0.565 (0.449,0.662)	0.586
ADC(×10^-3^mm2/s)	Mean	0.79 ± 0.13	0.92 ± 0.11	0.961 (0.952,0.973)	0.946 (0.937,0.963)	0.502
	25th	0.71 ± 0.13	0.83 ± 0.10	0.927 (0.913,0.942)	0.919 (0.907,0.926)	0.189
	50th	0.78 ± 0.13	0.89 ± 0.10	0.947 (0.931,0.954)	0.943 (0.925,0.949)	0.166
	75th↵	0.86 ± 0.15	0.98 ± 0.14	0.936 (0.925,0.943)	0.929 (0.917,0.938)	0.488
	100th	1.13 ± 0.21	1.30 ± 0.27	0.723 (0.648,0.791)	0.642 (0.594,0.796)	0.161
	Skewness	0.66 ± 0.71	0.76 ± 0.89	0.626 (0.571,0.748)	0.721 (0.682,0.829)	0.313
	Kurtosis	3.88 ± 1.76	4.59 ± 3.30	0.512 (0.472,0.675)	0.465 (0.419,0.593)	0.390
Ss-EPI MK	Mean	1.12 ± 0.20	0.97 ± 0.15	0.945 (0.925,0.959)	0.922 (0.894,0.942)	0.032
	25th	1.00 ± 0.20	0.88 ± 0.13	0.943 (0.923,0.958)	0.931 (0.907,0.949)	0.075
	50th	1.11 ± 0.18	0.96 ± 0.13	0.933 (0.910,0.951)	0.945 (0.926,0.960)	0.032
	75th	1.22 ± 0.22	1.07 ± 0.17	0.915 (0.886,0.937)	0.911 (0.880,0.934)	0.044
	100th	1.69 ± 0.89	1.38 ± 0.43	0.822 (0.764,0.866)	0.465 (0.335,0.578)	0.053
	Skewness	0.15 ± 1.07	0.10 ± 1.13	0.732 (0.651,0.780)	0.512 (0.388,0.618)	0.332
	Kurtosis	4.57 ± 3.09	4.49 ± 2.87	0.629 (0.525,0.714)	0.367 (0.225,0.493)	0.986
MD(×10^-3^mm2/s)	Mean	1.07 ± 0.23	1.24 ± 0.21	0.941 (0.921,0.957)	0.949 (0.931,0.962)	0.940
	25th	0.92 ± 0.21	1.08 ± 0.17	0.943 (0.923,0.958)	0.955 (0.939,0.967)	0.397
	50th	1.04 ± 0.23	1.21 ± 0.20	0.984 (0.979,0.989)	0.961 (0.947,0.971)	0.395
	75th	1.18 ± 0.26	1.38 ± 0.25	0.965 (0.953,0.974)	0.967 (0.956,0.976)	0.958
	100th	1.69 ± 0.42	1.98 ± 0.52	0.765 (0.692,0.822)	0.732 (0.651,0.797)	0.176
	Skewness	0.77 ± 0.87	0.89 ± 0.89	0.773 (0.702,0.828)	0.724 (0.641,0.790)	0.484
	Kurtosis	4.24 ± 2.54	4.58 ± 2.74	0.599 (0.489,0.690)	0.676 (0.582,0.752)	0.989
ADC(×10^-3^mm2/s)	Mean	0.71 ± 0.14	0.83 ± 0.14	0.917 (0.879,0.935)	0.905 (0.861,0.915)	0.995
	25th	0.64 ± 0.13	0.75 ± 0.12	0.875 (0.824,0.901)	0.798 (0.771,0.811)	0.599
	50th	0.70 ± 0.14	0.82 ± 0.13	0.891 (0.881,0.915)	0.885 (0.867,0.897)	0.646
	75th	0.77 ± 0.15	0.91 ± 0.16	0.904 (0.875,0.922)	0.873 (0.841,0.892)	0.963
	100th	1.02 ± 0.23	1.18 ± 0.33	0.712 (0.624,0.857)	0.636 (0.557,0.746)	0.192
	Skewness	0.54 ± 0.92	0.63 ± 0.86	0.534 (0.421,0.626)	0.684 (0.572,0.778)	0.332
	Kurtosis	4.07 ± 2.10	4.18 ± 1.96	0.506 (0.447,0.649)	0.465 (0.419,0.593)	0.358

BC, breast cancer; ICC, intraclass correlation coefficients; Cl, confidence interval; MK, mean kurtosis; MD, mean diffusion; ADC, apparent diffusion coefficient; ss-EPl, single-shot echo-planar imaging; rs-EPl, readout-segmented echo planar imagin.

**Table 2:** Histogram parameters between ss-EPl and ts-EPI sequence for luminal and non-luminal BC.

Variables	Luminal BC			Non-luminal BC		
	ss-EPI	rs-EPI	*P*	ss-EPI	rs-EPI	*P*
Histogram MK						
Mean	1.12 ± 0.20	0.96 ± 0.16	0.014	0.97 ± 0.15	0.80 ± 0.08	0.000
25th	1.00 ± 0.20	0.85 ± 0.16	0.079	0.88 ± 0.13	0.72 ± 0.10	0.047
50th	1.11 ± 0.18	0.96 ± 0.16	0.045	0.96 ± 0.13	0.81 ± 0.08	0.023
75th	1.22 ± 0.22	1.08 ± 0.20	0.044	1.07 ± 0.17	0.88 ± 0.07	0.020
100th	1.69 ± 0.89	1.46 ± 0.56	0.099	1.38 ± 0.43	1.11 ± 0.22	0.413
skewness	0.15 ± 1.07	0.02 ± 1.10	0.759	0.10 ± 1.13	-0.28 ± 1.13	0.455
kurtosis	4.57 ± 3.09	4.60 ± 4.14	0.504	4.49 ± 2.87	4.92 ± 4.13	0.532
MD(×10^-3^mm2/s)						
Mean	1.07 ± 0.23	1.12 ± 0.20	0.263	1.24 ± 0.21	1.26 ± 0.29	0.399
25th	0.92 ± 0.21	0.98 ± 0.19	0.297	1.08 ± 0.17	1.11 ± 0.17	0.901
50th	1.04 ± 0.23	1.09 ± 0.20	0.195	1.21 ± 0.20	1.22 ± 0.27	0.446
75th	1.18 ± 0.26	1.23 ± 0.23	0.182	1.38 ± 0.25	1.38 ± 0.21	0.194
100th	1.69 ± 0.42	1.78 ± 0.38	0.245	1.98 ± 0.52	1.98 ± 0.45	0.460
skewness	0.77 ± 0.87	0.80 ± 0.69	0.279	0.89 ± 0.89	0.85 ± 0.72	0.440
kurtosis	4.24 ± 2.54	4.11 ± 1.89	0.435	4.58 ± 2.74	4.25 ± 1.67	0.051
ADC(×10^-3^mm2/s)						
Mean	0.71 ± 0.14	0.79 ± 0.13	0.464	0.83 ± 0.14	0.92 ± 0.11	0.741
25th	0.64 ± 0.13	0.71 ± 0.13	0.550	0.75 ± 0.12	0.83 ± 0.10	0.640
50th	0.70 ± 0.14	0.78 ± 0.13	0.517	0.82 ± 0.13	0.89 ± 0.10	0.139
75th	0.77 ± 0.15	0.86 ± 0.15	0.547	0.91 ± 0.16	0.98 ± 0.14	0.368
100th	1.02 ± 0.23	1.13 ± 0.21	0.239	1.18 ± 0.33	1.30 ± 0.27	0.589
skewness	0.54 ± 0.92	0.66 ± 0.71	0.422	0.63 ± 0.86	0.76 ± 0.89	1.000
kurtosis	4.07 ± 2.10	3.88 ± 1.76	0.364	4.18 ± 0.96	4.59 ± 3.30	0.245

BC, breast cancer; MK, mean kurtosis; MD, mean diffusion; ADC, apparent diffusion coefficient; ss-EPl, single-shot echo-planar imaging; rs-EPI, readout-segmented echo planar imaging.

### Diagnostic accuracy of Histogram metrics between ss-EPI and rs-EPI sequence

**Figure 3:**
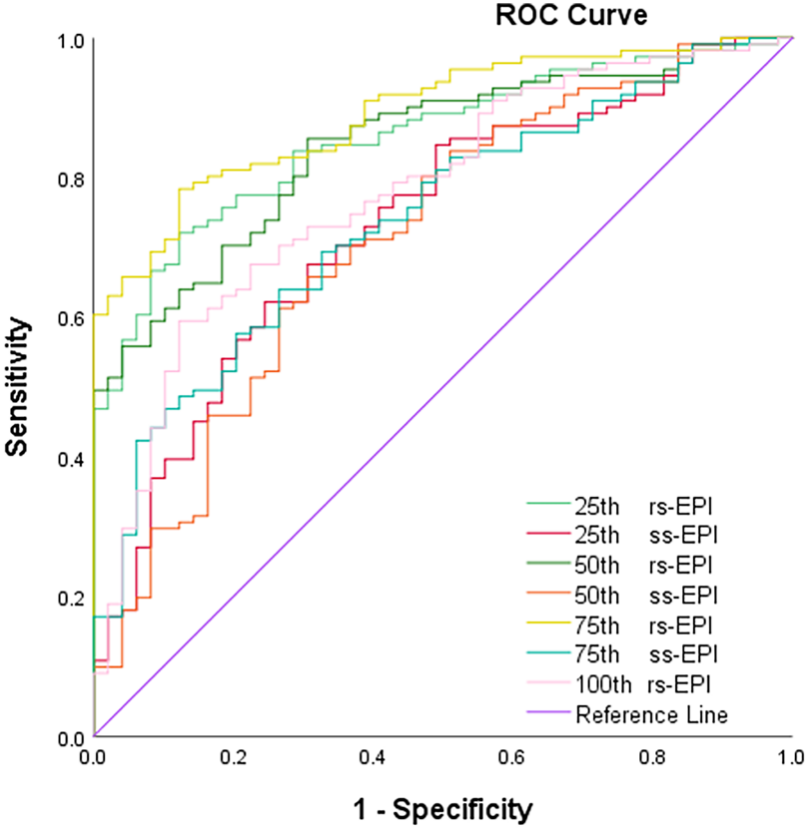
Receiver operating characteristic curves of significant histogram metrics of both sequences in distinguishing luminal from non-luminal breast cancer.

The ROC curve analysis for significant histogram metrics of both sequences in distinguishing luminal from non-luminal BC is displayed in Table 3 and Figure 3. Analyses for ROC curves showed that MK_mean_, MK_50th_, MK_75th_ and MK_100th_ derived from rs-EPI yielded AUCs of 0.857, 0.847, 0.891, and 0.778. MK_mean_, MK_50th_ and MK_75th_ derived from ss-EPI yielded AUCs of 0.733, 0.714, and 0.740. Figure 3 showed the AUC comparisons of significant histogram metrics in differentiation of luminal vs. non-luminal BC. MK75th derived from rs-EPI yielded the highest AUC of 0.891, with the sensitivity of 78.4%, the specificity of 87.8%, and a cutoff value of 0.966 × 10^-3^ for distinguishing luminal from non-luminal BC.

**Table 3:** ROC analyses of significant histogram metrics in differentiating luminal from non-luminal BC between ss-EPI and rs-EPI sequence.

	Variables	Youden Index	Cutoff Value	Sensitivity (%)	Specificity (%)	AUC	95%CI
rs-EPI	MK						
	Mean	0.599	> 0.882	72.1	87.8	0.857	0.793–0.907
	50th	0.55	> 0.845	85.6	69.4	0.847	0.781–0.899
	75th	0.662	> 0.966	78.4	87.8	0.891	0.832–0.935
	100th	0.473	> 1.240	59.5	87.8	0.778	0.706–0.840
ss-EPI	MK						
	Mean	0.377	> 1.039	62.2	75.5	0.733	0.657–0.800
	50th	0.352	> 1.041	65.8	69.4	0.714	0.638–0.783
	75th	0.375	> 1.115	64	73.5	0.74	0.665–0.806

MK, mean kurtosis; MD, mean diffusion; ADC, apparent diffusion coefficient; ss-EPl, single-shot echo-planar imaging; rs-EPI, readout-segmented echo planar imaging. AUC, area under the curve.

## Discussion

In our study, histogram metrics of DKI and DWI parameters for distinguishing between luminal and non-luminal BC were compared between ss-EPI and rs-EPI sequence. The findings of this study showed that mean values, 50th/75th percentiles of MK derived from both sequences and 100 percentiles of MK derived from rs-EPI were able to distinguish luminal and non-luminal BC. Moreover, MK histogram parameters derived from rs-EPI significantly improved diagnostic performance for characterization of luminal versus non-luminal BC in comparison with that derived from ss-EPI. MK_75th_ derived from rs-EPI had the largest AUC in discriminating luminal from non-luminal BC. To our knowledge, there was no previous study to compare the diagnostic performances of DKI and DWI histogram metrics between ss-EPI and rs-EPI for differentiating luminal from non-luminal BC.

Luminal BC are more commonly observed in patients diagnosed with invasive BC and have more favorable prognosis compared with non-luminal BC^2^. Previous studies have attempted to investigate the value of DWI in differentiating luminal and non-luminal BC, but the highest AUC of DWI quantitative parameters varied from 0.608 to 0.710^20,22,31^. The imaging protocols for DWI acquisition and DWI models for quantitative parameters calculation adapted in these studies are not consistent. Thus, we explored the baseline techniques for DWI acquisition including single-shot and multi-shot echo-planar imaging (EPI) (ss-EPI vs. rs-EPI) in the differentiation of luminal vs. non-luminal BC. This research showed that the luminal subtype showed a higher MK value (MK_mean_, MK_50th_ and MK_75th_) compared to non-luminal subtype in both DWI sequences. The findings were consistent with previous findings^21,22^. Hormone receptor (ER/PR) overexpression could inhibit tumors angiogenic pathways and therefore results in reduced blood perfusion and increase cellularity to restrict water diffusion^31,32^. In addition, the more characteristic morphological feature of luminal BC is having spiculated margin, which might be correlated with tumor infiltration and microstructural heterogeneity^33,34^. Thus, higher kurtosis values in luminal BC may be partially explained by the above reasons. However, all histogram metrics of MD and ADC failed to provoke significant differences between the luminal and non-luminal BC in our study. These results were in line with a study investigated by Kang et al.^22^. The reason for it is that diffusion value (MD and ADC) measurements might be greatly affected by partial-volume effects from uneven fat suppression under high b value (1000 s/mm2) imaging^25,35^. What’s more, MK_75th_ showed the highest AUC among all histogram metrics of MK for differentiating luminal from non-luminal BC in both ss-EPI and rs-EPI. Due to the histologic heterogeneity of BC, the mean value of MK may underestimate the the deviation of tissue diffusion and the higher percentiles of MK may reflect the most aggressive components of BC except MK_100th_^14^. Besides, MK_100th_ did not exhibit the highest AUC because the maximum MK value might be more susceptible to noise and adjacent structures^29,36^.

There existed significant differences in some histogram metrics of MK between two sequence for luminal and non-luminal BC. The non-luminal BC showed statistically significant differences in the MK_mean_, MK_25th_, MK_50th_ and MK_75th_ between ss-EPI and rs-EPI sequence. The ROC analyses also suggest that the diagnostic performance of each histogram metric of MK in rs-EPI sequence was better than the respective histogram metric in ss-EPI sequence. As is known to all, ss-EPI is widely applied for breast DWI. However, the main challenges for ss-EPI are gradient nonlinearities, susceptibility-induced geometric distortion and motion artifacts due to phase error accumulation during the long EPI readout^37^. These defects lead to inaccuracies in quantitative parameters calculation and degrade the performance of DWI quantitative parameters^38^. The rs-EPI sequence can shorten echo train lengths with parallel imaging techniques and improve the deficiencies faced with ss-EPI sequence, particularly at 3.0-T^39^.

## Limitations

Our study presents some limitations. First, this was a prospective study based in a single center, with a relatively small sample size, especially in non-luminal BC. Multicenter cohort studies would also be needed to further validate our findings. Second, the lesions included in our study were mass lesions. It would be helpful to explore whether there exist some differences between mass and non-mass like lesions and we will explore it in the future. Third, all MRI acquisitions were performed on a single MRI unit and analyzed using one prototype software, the generalizability of our findings may be limited. What’s more, the two-dimensional ROIs were manually defined, which may be insufficient to reveal the whole-tumor heterogeneity. Whole-tumor histogram analysis of quantitative parameters may better reflect the intratumoral heterogeneity of BC.

## Conclusion

The rs-EPI sequence improves the diagnostic accuracy of the differentiation between luminal and non-Luminal breast cancer. Histogram analysis may be useful for differentiating luminal from non-luminal BC. The 75th percentile of MK derived from the rs-EPI sequence was the most valuable metric among all the histogram metrics.

## Data Availability

The datasets used during the current study is available from the corresponding author on reasonable request.
